# Explainable AI-driven assessment of hydro climatic interactions shaping river discharge dynamics in a monsoonal basin

**DOI:** 10.1038/s41598-025-13221-x

**Published:** 2025-07-26

**Authors:** Prashant Parasar, Akhouri Pramod Krishna

**Affiliations:** Department of Remote Sensing, Birla Institute of Technology, Mesra, Ranchi, Jharkhand 835215 India

**Keywords:** Kolmogorov Arnold networks, Explainable artificial intelligence, River discharge forecasting, CMIP6, SHAP interpretability, Subarnarekha river basin, Climate change, Hydrology

## Abstract

Accurate river discharge forecasting is essential for effective water resource management, particularly in regions prone to monsoonal variability and extreme weather events. This study presents an interpretable deep learning framework for daily river discharge forecasting in the Subarnarekha river basin (SRB), integrating Kolmogorov Arnold networks (KAN) with Shapley additive exPlanations (SHAP). Leveraging hydroclimatic inputs from five coupled model intercomparison project phase 6 (CMIP6) general circulation models (GCM) under the high emissions shared socioeconomic pathway (SSP585) scenario, the model was trained and evaluated across four active gauging stations: Muri, Adityapur, Jamshedpur, and Ghatsila covering the period 1980 to 2022, with projections extending to 2100. The main findings of this study are (1) KAN demonstrated high predictive performance with root mean squared error (RMSE) values ranging from 42.7 to 58.3 m^3^/s, Nash–Sutcliffe efficiency (NSE) between 0.80 and 0.87, mean absolute error (MAE) between 28.9 to 52.7 and R^2^ values between 0.84 and 0.90 across stations. (2) SHAP based feature contribution analysis identified Relative humidity *(hurs)*, specific humidity *(huss)*, and temperature *(tas)* as key predictors, while (*pr)* showed limited contribution due to spatial inherent inconsistencies in GCM precipitation data. (3) The bootstrapped SHAP distributions highlighted substantial variability in feature importance, particularly for humidity variables, revealing station specific uncertainty patterns in model interpretation. (4) The KAN framework results indicate strong temporal alignment and physical realism, confirming KAN’s robustness in capturing seasonal discharge dynamics and extreme flow events under monsoon influence environments. (5) In this study KAN with SHAP (SHapley additive exPlanations) is implemented for hydrological modeling under monsoon-influenced and data-limited regions such as SRB, offering improved accuracy, functional precision and efficiency compared to traditional models. The explainability offered by SHAP confirms informed water resource planning. This novel framework presents a reproducible and climate-resilient decision support tool, particularly suitable for monsoon-influenced, data-limited basins susceptible to extreme hydroclimatic events.

## Introduction

Reliable and accurate river discharge forecasting is critical for sustainable water resource management under changing climatic conditions^1^. Monsoon-dominated regions such as the SRB may even be more challenging. Since such regions are typified by highly variable seasonal precipitation, limited observational infrastructure, and complex topographic features. All of these contribute to nonlinear and dynamic hydrological responses^2–5^. River discharge forecast is essential for a wide application, including reservoir operations, watershed planning, flood mitigation, agricultural planning, and ecosystem conservation^6–10^. However, physically based traditional hydrological models sometimes encounter limitations. These models typically require high spatial resolution data inputs and involve massive calibration requirements, which may be challenging to implement in data-scarce environments^11–15^.

However, in recent years, climate change has had a profound impact on both the groundwater and surface water systems, altering the natural balance of hydrologic fluxes^16,17^. These disruptions have intensified the frequency and severity of natural disasters, including floods^18^ and droughts^19^, while also affecting the operational stability of dams and reservoirs^20^. Additionally, climate-induced changes have negatively influenced agricultural productivity^21^, degraded water quality, and undermined the resilience of sustainable livelihoods, particularly in vulnerable regions^22^.

Data-driven approaches, particularly machine learning (ML), have gained traction in hydrological modeling for their ability to learn complex, nonlinear patterns directly from empirical data without relying on explicit physical formulations^10^. Models such as random forest (RF), support vector regression (SVR), and deep learning approaches like long short-term memory (LSTM) networks have demonstrated considerable improvements in river discharge forecasting across diverse hydroclimatic contexts^8,23–26^. However, these models have a significant limitation of black box nature, restricting interpretability, which hinders their practical adoption extensively. To overcome the interpretability deficit, Explainable Artificial Intelligence (XAI) techniques such as Shapley Additive exPlanations (SHAP)^27^ have emerged as effective tools for providing insight into model behaviour and feature attribution^28–32^. SHAP offers both global and local interpretability, enabling a nuanced understanding of how individual predictors influence model outputs. Recent hydrological research has increasingly adopted SHAP to enhance the transparency and diagnostic capabilities of ML-based discharge prediction frameworks^33–38^.

Despite these advancements, most existing ML applications in hydrology rely on conventional architectures such as ensemble trees and Multilayer Perceptrons (MLPs), which lack structural flexibility and offer limited theoretical foundations for functional interpretation^39–43^. While models like RF, SVR and LSTM have been previously applied in the SRB and have improved predictive accuracy. However, these models still function as black boxes, offering limited insights into hydrological causality. Although attention-based architecture^44–46^ have been introduced to enhance temporal learning in river discharge prediction, their interpretability remains constrained.

To address these gaps, KANs have recently been introduced as a novel neural architecture grounded in the Kolmogorov-Arnold representation theorem. This theorem asserts that any multivariate continuous function. Unlike MLPs that exist on each node, KANs employ learnable spline-based univariate transformations along network edges, which correspond to the weights of a traditional neural network, and every edge has a different activation function, resulting in more interpretable, adaptive, and efficient functional approximations. Empirical studies have demonstrated that KANs can achieve superior performance with significantly fewer parameters compared to traditional deep neural networks, particularly in solving complex, high-dimensional problems such as partial differential equations and dynamic systems modeling^47–49^. Replacing dilated convolutions and attention mechanisms with spline-based KANs, which inherently capture nonlinear dependencies without requiring elaborate architectural choices.

By replacing dilated convolutions and attention mechanisms with spline-based KANs, which inherently capture nonlinear dependencies without requiring elaborate architectural tuning^48,50^. Their structural efficiency, theoretical grounding, and adaptability to sparse data contexts make them especially appropriate for monsoon-influenced or data-limited basins like SRB. Furthermore, when coupled with SHAP, KANs form a transparent and theoretically robust modeling framework capable of elucidating the physical mechanisms underpinning discharge variability. This study introduces a novel modeling framework that combines strengths of KAN with SHAP-based interpretability for daily river discharge forecasting. To our knowledge, this is the first application of KAN in combination with SHAP in a hydrological context, particularly within monsoon-influenced or data-limited regions such as the SRB. The proposed framework ensures not only high predictive performance but also enhanced interpretability. In contrast to existing models such as TeaNet^45^, which primarily utilize (*tas)* and (*pr)* as predictors, the proposed approach integrates additional atmospheric variables, including (*hurs),* and (*huss)*, which have been identified as key drivers of streamflow variability. This comprehensive input selection enables a more nuanced characterization of hydrological responses.

## Material and methodology

### Study area

**Fig. 1:**
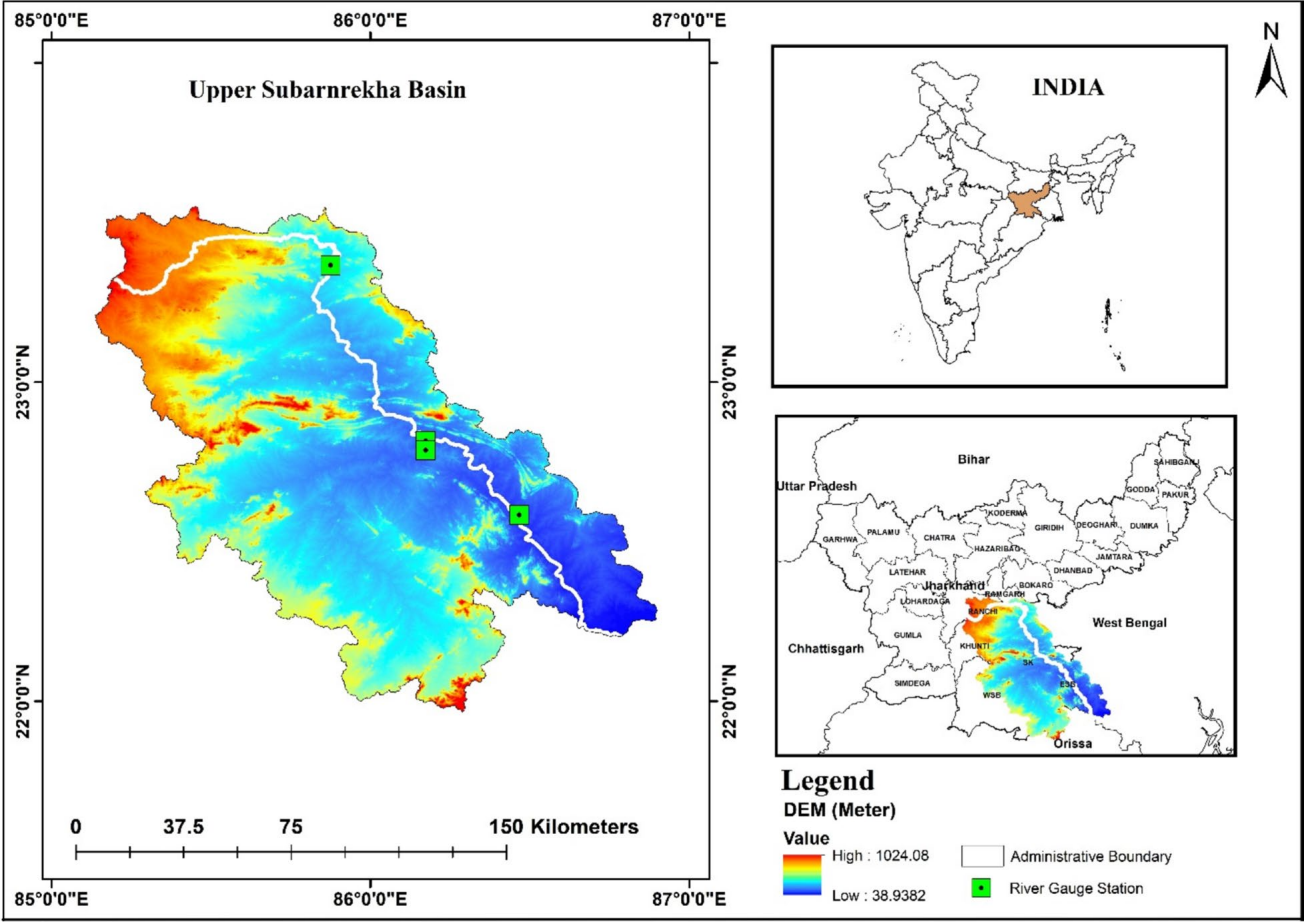
Location map of the active river gauge station within the study area. The Map created using ArcMap v.10.3.

SRB, situated in eastern India, spans the states of Jharkhand, West Bengal, and Odisha. Originating near Nagri village in Ranchi district at an elevation of approximately 610 m, the river flows for about 395 km before joining the Bay of Bengal. The basin extends over an area of approximately 19,300 km^2^ and lies between 21°30′N to 23°30′N latitudes and 85°00′E to 87°30′E longitudes. Geomorphologically (Fig. 1), it is a part of the Chotanagpur plateau, which is characterized by rugged topography, moderate slopes, lateritic and red soils, and significant forest cover in its upper reaches^51,52^. The climate of the region is tropical monsoonal, with an average annual precipitation between 1200 and 1600 mm^53^, most of which falls during the southwest monsoon (June–September). Mean monthly temperatures range from about 10° c in January to over 35° c in May. This pronounced seasonal variation in precipitation and temperature significantly affects the basin hydrology, resulting in flash floods during intense monsoon spells and low flow conditions during dry periods^54^.

Hydrologically, the SRB is a rain-fed river system, making it highly sensitive to precipitation variability and climate change. The basin contains several small to medium-scale reservoirs but lacks major dam infrastructure. Urban industrial hubs such as Ranchi, Jamshedpur and Adityapur exert significant anthropogenic pressure, leading to altered flow regimes and water quality degradation^52,55,56^. This study focuses on four key hydrological stations, Muri, Adityapur, Jamshedpur, and Ghatsila, which capture spatial variability in climate, land use, and terrain. These locations provide a representative basis for developing robust and interpretable machine learning models for river discharge forecasting in monsoon-influenced or data-limited regions.

### Data source and preprocessing

#### Hydroclimatic and gauge station data

Data from four active river gauge stations of the SRB, namely Muri, Jamshedpur, Adityapur and Ghatsila, obtained from the central water commission (CWC), were employed in this study comprises daily scale gauge station river discharge records spanning from 1980 to 2022 and a suite of hydroclimatic variables extending from 1 January 1980 to 31 December 2100. The hydroclimatic dataset integrates both historical observations and future projections derived from the Coupled Model Intercomparison Project Phase 6 (CMIP6). Climate projections were obtained under the high emission Shared Socioeconomic Pathway scenario (SSP585), ensuring a consistent framework for evaluating long-term hydrological responses to climatic variability (Table 1).

**Table 1:** Specifications of the selected CMIP6 models used for discharge prediction under the SSP 585 scenario.

Model name	Modeling center	Country	resolution (atmosphere)	Data availability	
				(GCM models)	(Gauge-station)
ACCESS-CM2	CSIRO-ARCCSS	Australia	~ 1.875° × 1.25°	1980–2100	1980–2022
CNRM-CM6-1	CNRM-CERFACS	France	~ 1.4° × 1.4°	1980–2100	1980–2022
MIROC6	MIROC (Japan)	Japan	~ 1.4° × 1.4°	1980–2100	1980–2022
MRI-ESM2-0	Meteorological Research Institute	Japan	~ 1.125° × 1.125°	1980–2100	1980–2022
CanESM5	Canadian Centre for Climate Mod	Canada	~ 2.81° × 2.81°	1980–2100	1980–2022

Specifically, five well established CMIP6 GCM were used: ACCESS-CM2, CNRM-CM6-1, MIROC6, MRI-ESM2-0, and CanESM5. These models were selected based on their spatial resolution, availability of essential hydroclimatic variables, and documented performance in simulating South Asian monsoon dynamics. The GCM data were acquired from the Earth System Grid Federation (ESGF) data portal (https://www.nccs.nasa.gov/services/data-collections/land-based-products/nex-gddp cmip6), a globally recognized repository for CMIP6 datasets. Predictor variables used in the analysis included precipitation (pr), mean temperature (tas), maximum and minimum temperatures (tasmax, tasmin), relative humidity (hurs), and specific humidity (huss), representing key atmospheric controls over hydrological processes.

Before modeling, datasets were examined for missing values. Minor missing values in both discharge and meteorological time series were filled using linear interpolation to preserve temporal continuity. This ensured a complete and consistent dataset for model development. The datasets were split into (70%) training and (30%) testing sets using a fixed random seed of 42, a widely adopted standard in machine learning studies. The 70/30 division was selected as it represents a conventional and balanced approach for training and evaluating model performance while avoiding overfitting. All data preprocessing, statistical bias correction, and evaluation were conducted in Google Collaboratory (Colab), a cloud-based Python development environment. The implementation was performed using Python version 3.10.12, and key scientific libraries included xarray for multidimensional array processing, pandas for time series manipulation, scikit learn for ML model development, and SHAP for model interpretability. Furthermore, all spatial maps, including basin boundaries and station locations, were prepared using ArcMap 10.3 (ESRI Inc., USA).

#### Preprocessing and Bias correction of GCM Data

**Fig. 2:**
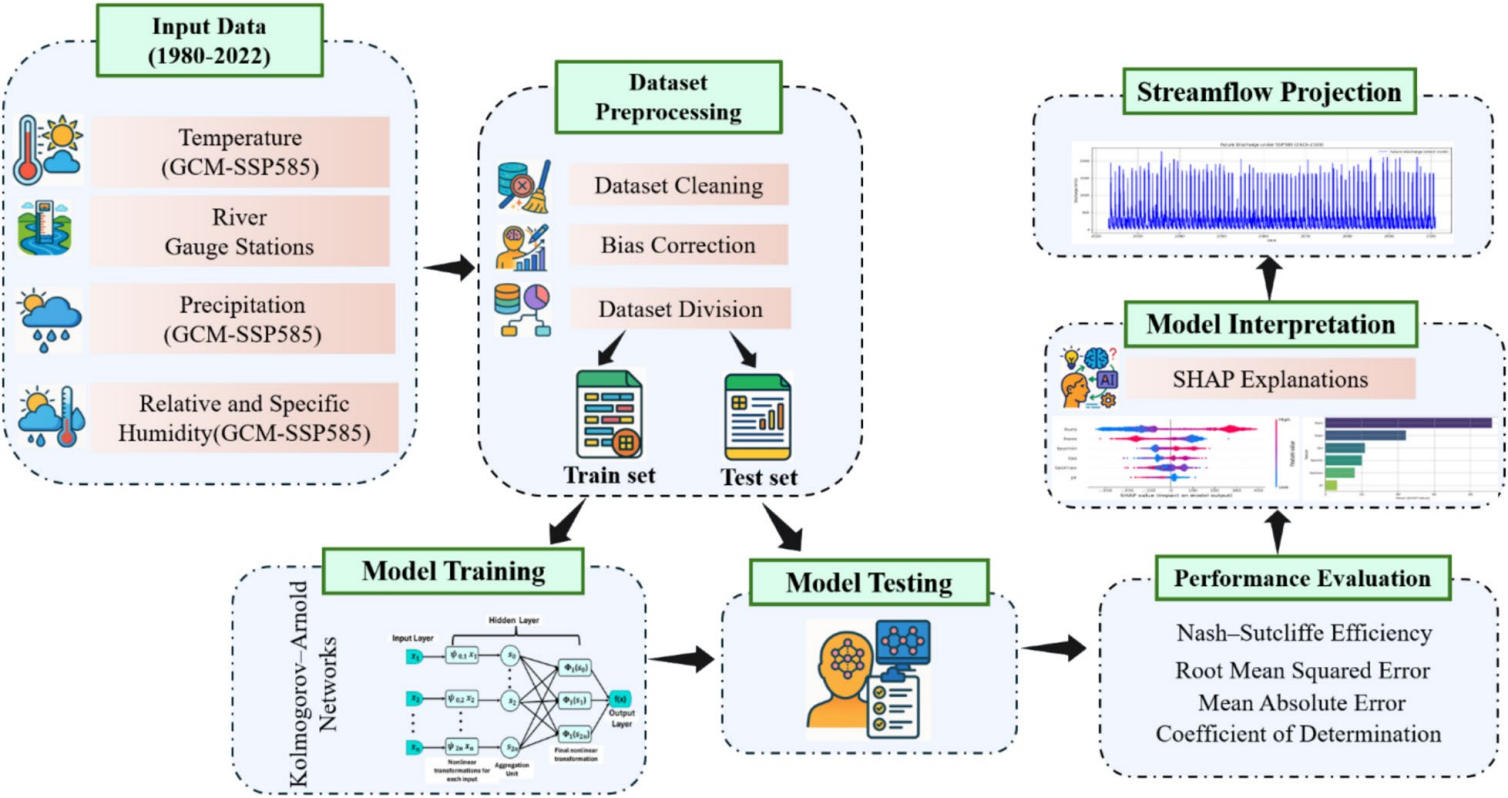
Workflow of the KAN with SHAP framework for discharge prediction.

GCMs are essential for projecting future hydroclimatic conditions under different emission scenarios. However, their raw outputs often exhibit systematic biases when compared to observed or reanalysis datasets due to coarse spatial resolution, simplifications in land atmosphere interactions, and errors in convective parameterizations^57^. These biases must be corrected before using GCM outputs for hydrological modeling, particularly for river discharge prediction, to ensure realistic magnitudes, distribution shapes, and temporal coherence. The target variable is daily river discharge (m^3^/s). All temperature variables originally expressed in Kelvin were converted to degrees Celsius to align with hydrological standards. In this study, a multi-step bias correction procedure was employed to improve the fidelity of CMIP6 model outputs from ACCESS-CM2, CNRM-CM6-1, MIROC6, MRI-ESM2-0, and CanESM5 under the SSP585 scenario. The procedure includes data harmonization, statistical correction, and scenario extension, as described below in (Fig. 2).

#### Delta change method (DCM)

The delta change method is a widely used technique for future scenario extension. It adjusts baseline observed data using the relative or absolute change projected by GCMs. In this study, absolute delta changes were applied to temperature variables, while relative changes were applied to precipitation, aligning with standard practice in hydrological modeling to maintain physical consistency. It ensures the consistency with historical observations while preserving the future climate projections signal^58^.

For additive variables, such as temperature, is given by Eq. (1):

1 $${T}_{\text{future }}^{\text{corrected }}\left(t\right)={T}_{\text{obs }}\left(t\right)+\left({T}_{\text{GCM},\text{ future }}\left(t\right)-{T}_{\text{GCM},\text{ hist }}\left(t\right)\right)$$

For multiplicative variables such as precipitation, as shown in Eq. (2):

2 $${P}_{\text{future }}^{{\text{correcte}}\text{d }}\left(t\right)={P}_{\text{obs}}\left(t\right)\times \frac{{P}_{\text{GCM},\text{ future }}\left(t\right)}{{P}_{\text{GCM},\text{ hist }}\left(t\right)}$$

where:

- ${T}_{\text{obs }}(t),{P}_{\text{obs }}(t)$ are the observed baseline data,
- ${T}_{\text{GCM}}$, future, ${P}_{\text{GCM}}$, future are raw future GCM outputs,
- ${T}_{\text{GCM}}$, hist, ${P}_{\text{GCM},\text{ hist}}$ are historical GCM runs (1980–2014).

#### Quantile mapping (QM)

Empirical quantile mapping (EQM) corrects discrepancies between observed and GCM distributions by matching percentiles. Precipitation was corrected using gamma distribution fitting, and temperature using normal distribution, as per^59–61^. In this study, 100 quantiles are used to construct empirical cumulative distribution functions, ensuring high-resolution mapping across the data range. This level of granularity allows for percentile-level correction of biases, which is particularly important for capturing extremes in precipitation and temperature while maintaining computational efficiency and reliability. Gamma distribution fitting was used for precipitation, as it appropriately captures the skewed nature of rainfall, while normal distribution was used for temperature variables, which generally follow symmetric distributions. This ensures alignment in probability distributions across datasets, maintaining both shape and central tendency.

Let: ${F}_{\text{obs }}^{-1}$ be the empirical inverse CDF of observed data,

${F}_{GCM}$ be the CDF of GCM data,

X be a simulated GCM value.

The corrected value is:

3 $${x}^{\text{corrected }}={F}_{\text{obs }}^{-1}\left({F}_{\text{GCM}}(x)\right)$$

This correction ensures that:

- The GCM data follow the observed distribution during the baseline period,
- Bias-corrected projections retain the statistical properties of historical records while incorporating future shifts.
- We used gamma distribution fitting for precipitation and normal distribution fitting for temperature, following best practices outlined in^62,63^

#### Linear scaling (LS)

LS adjusts long-term GCM means to match observed climatology without altering intra-annual variability. It was primarily applied to temperature variables in this study. The simplicity and computational efficiency of LS make it a practical choice for large-scale hydrological studies, particularly when model transparency is a priority. Recent research^45,64,65^ has reaffirmed the utility of LS in monsoon-driven catchments, where seasonal consistency and mean alignment are more critical than variance preservation. Although LS does not correct for higher-order statistical moments, it is still widely recommended as a first-pass bias correction method due to its ease of implementation and reproducibility across CMIP6 datasets.

For comparative analysis, a linear scaling (LS) method was also applied for temperature correction using Eq. (4):

4 $${T}^{\text{corrected }}\left(t\right)={T}_{\text{GCM}}\left(t\right)+\left({\mu }_{\text{obs}}-{\mu }_{\text{GCM}}\right)$$

where: ${\mu }_{\text{obs}}$ and ${\mu }_{\text{GCM}}$, are the long-term means during the baseline period.

This ensures the GCM mean is aligned with observations without altering variance.

#### Kolmogorov Arnold networks model (KAN)

KAN is a novel neural architecture inspired by the *Kolmogorov-Arnold representation theorem*^48^, which provides a theoretical foundation for representing any multivariate continuous function as a finite composition of univariate functions. Traditional neural networks, such as *multilayer perceptrons (MLP)*, model functions through sequential linear transformations followed by nonlinear activations like *ReLU* or *sigmoid*. In contrast, *KANs* adopt a fundamentally different approach by directly implementing the theorem’s formulation. According to the KAN theorem, any continuous function $f\left({x}_{1},{x}_{2}\dots ,{x}_{n}\right)$ defined on a compact domain can be expressed as a finite sum of the form:

5 $$f\left({x}_{1},\dots ,{x}_{n}\right)=\sum_{q-0}^{2n} {\Phi }_{q}\left(\sum_{p=1}^{n} {\psi }_{q,p}\left({x}_{p}\right)\right)$$

Here, ${\psi }_{q,p}$ and ${\Phi }_{q}$ are continuous univariate functions. KAN Networks implement this structure by learning both inner functions ψ₍q,p₎ and outer functions ${\Phi }_{q}$, which are parameterized using B-splines, smooth and differentiable basis functions defined by a set of control points. In the KAN architecture, each input feature xₚ is passed through a set of univariate functions ${\psi }_{q,p}$, and the resulting values are summed to compute intermediate scalars:

6 $${s}_{q}= \sum_{p=1}^{n}\psi q,p{(x)}_{p}$$

where each ${\psi }_{q,p}$ is a learnable univariate function applied to input ${x}_{p}$. This intermediate scalar ${s}_{p}$ is then passed through the outer univariate function ${\Phi }_{q \left({s}_{q}\right)}$, which is also learned during training. These intermediate values are then passed through the outer functions:

$${y}_{q}={\Phi }_{q}{(s}_{q})$$

Finally, the model output is given by the aggregated sum:

7 $${f}_{(x)}= \sum_{q=0}^{2n}{y}_{a}$$

**Fig. 3:**
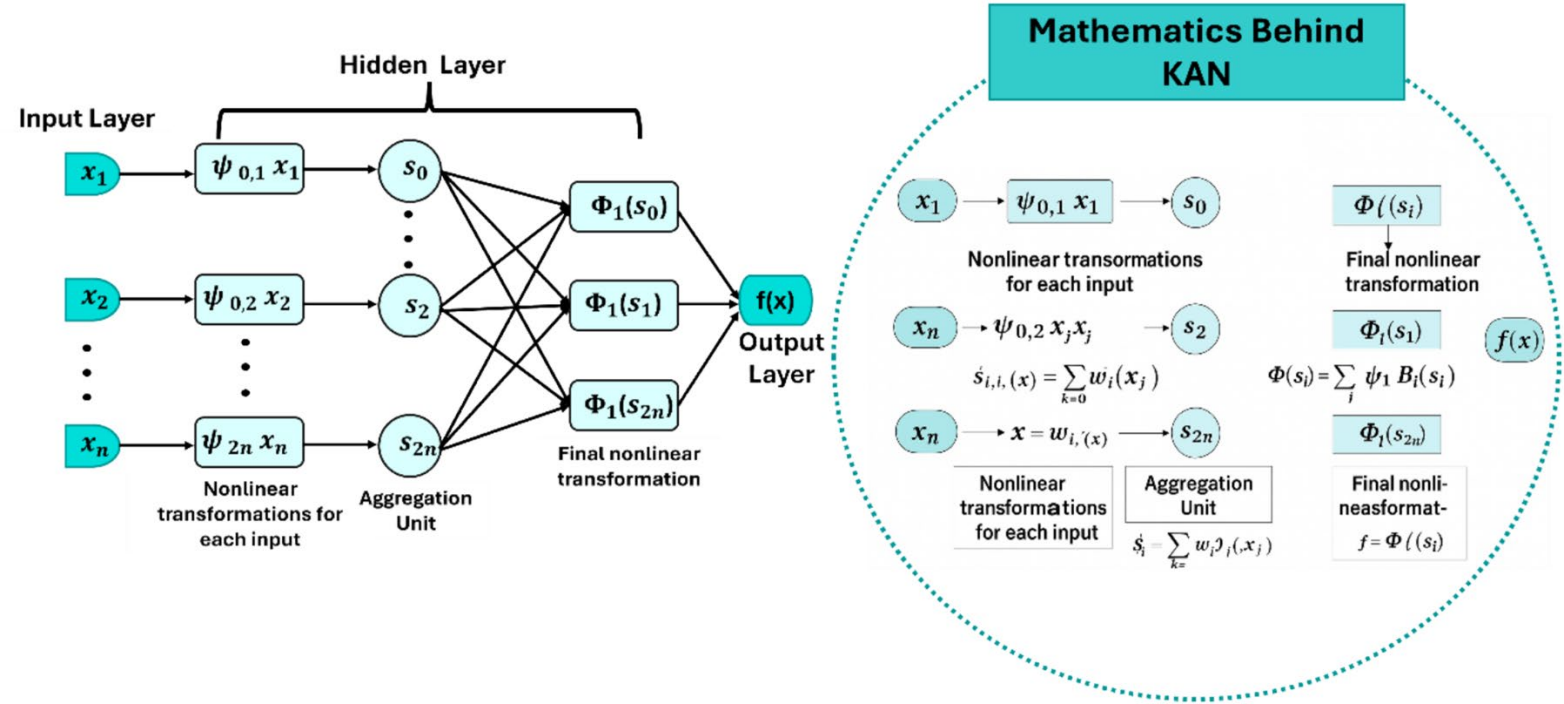
KAN architecture based on the KAN theorem, employing spline-based functional approximations for hydrological modeling.

To train the network, the spline control points defining the univariate functions are optimized using gradient descent. The training objective includes a loss function that measures prediction error on the training data, along with a regularization term to penalize excessive traditional neural architectures by shifting from weight-based learning to function-based learning. The structure of the KAN model is illustrated in (Fig. 3), which depicts its architecture based on the KAN theorem using spline-based functional approximations for hydrological modeling.

**Algorithm 1:**
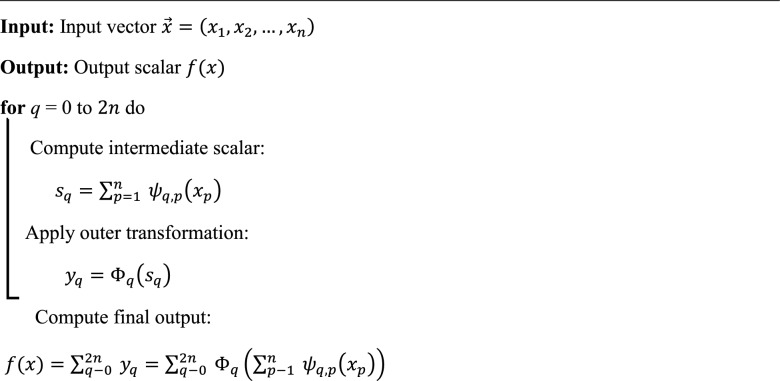
KAN.

#### Model interpretability through SHAP

To interpret the predictions of KAN, the SHAP framework was employed using KernelExplainer. Such an approach is increasingly growing focus in explainable AI. The use of the SHAP framework, particularly with model-agnostic tools like KernelExplainer, enables consistent and transparent estimation of feature contributions, regardless of the underlying model structure.

#### Interpretability and transparency of KANs

It is designed to be more interpretable than traditional deep learning models, allowing for the quantification and tracing of each computational step within the network. This transparency is a key advantage, making KANs suitable for practical deployment in fields where understanding models’ decisions is critical^66–68^. The ability of KANs to generate explicit mathematical formulas provides insights into the physical and mathematical dynamics underlying predictions, further supporting interpretability^67^.

#### SHAP framework for KAN interpretation

The SHAP framework, especially when using KernelExplainer, is model agnostic and can be applied to KANs to estimate feature contributions by approximating Shapley values. This ensures that the interpretability benefits of KANs are preserved and quantifiable, regardless of the model’s internal complexity^68^.

**Algorithm 2:**
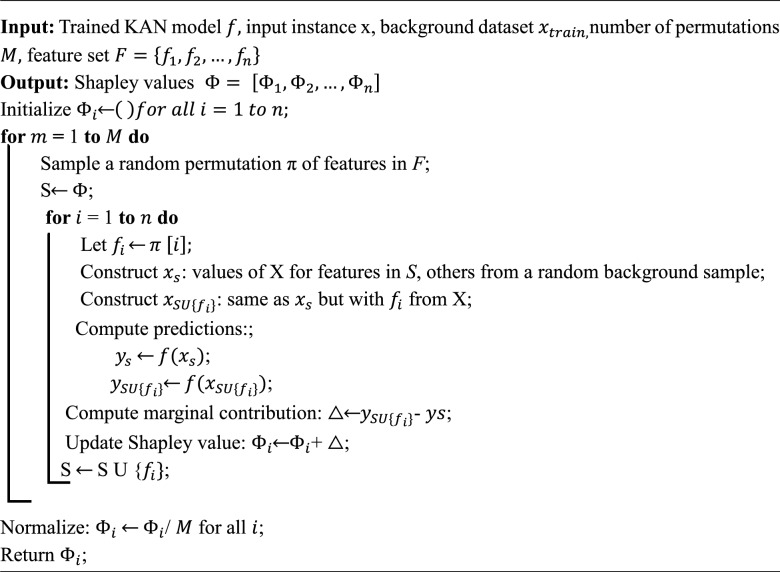
SHAP.

### Recent advances and applications

A summary of recent applications utilizing KAN and SHAP for explainability in environmental hydrological sciences is presented in (Table 2).

**Table 2:** Summary of recent applications integrating interpretability approaches with hydrological and environmental modeling.

Application area	Interpretability approach	Citations
Hybrid hydrological models	XAI for feature importance	^69^
LSTM streamflow models	Six XAI algorithms	^70^
Solar radiation & temperature	KAN + step quantification	^66^
Wind nowcasting	KAN + formula extraction	^67^
Time-series classification	KAN + SHAP analysis	^68^
Snowmelt-driven streamflow	Shapley and LIME variable importance	^71^

## Results and discussion

### Exploratory data analysis (EDA)

**Fig. 4:**
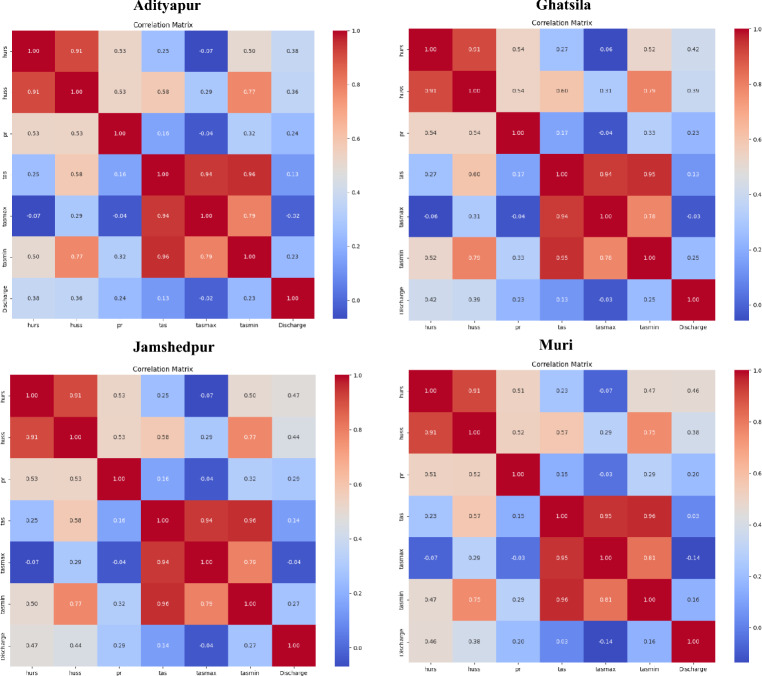
Correlation heatmaps of hydro-meteorological variables and river discharge for each gauge station.

**Fig. 5:**
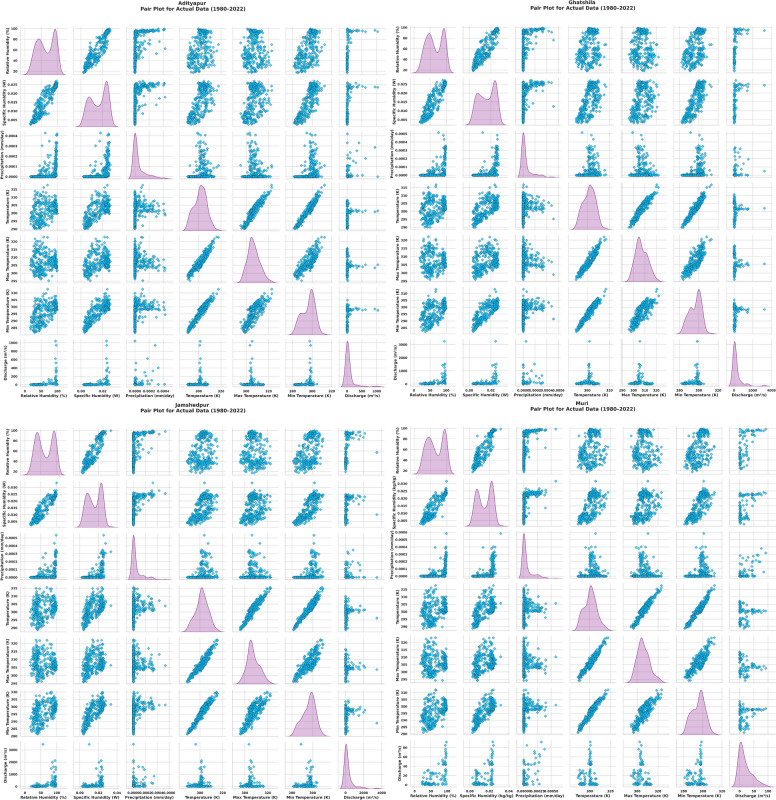
Pair plots of hydro-meteorological variables and river discharge for each gauge station.

EDA is an initial, informal examination of data aimed at discovering patterns, describing data, and formulating models using graphical and computational tools, which helps maximise the value of data, supports hypothesis development, and complements confirmatory data analysis^72,73^. As part of the EDA, correlation heatmaps and pair plots were generated for four key stations, Adityapur, Ghatsila, Jamshedpur and Muri, covering the period 1980 to 2022. The correlation matrices (Fig. 4) reveal strong positive interrelationships among temperature variables (*tas, tasmax, tasmin*; r ˃ 0.90) and between humidity variables (*hurs* and *huss*, r 0.91) across all stations. Discharge exhibits weak to moderate positive correlations with humidity, especially with huss at Ghatsila (r = 0.79), while its relationship with precipitation and temperature remains weak or negligible across sites. Pair plots (Fig. 5) further illustrate these trends, showing right-skewed discharge distributions and tightly clustered linear relationships among temperature variables. Scatter patterns between discharge and humidity or precipitation are diffuse, suggesting potential nonlinear interactions. Notably, discharge variability differs spatially, with broader ranges observed at Ghatsila and Jamshedpur, possibly due to differences in catchment characteristics or land use impacts. These findings emphasise the dominant influence of humidity and localised variability in discharge behaviour, offering important context for subsequent hydrological modeling.

### Multi-model ensemble of GCMs

GCMs are indispensable tools for projecting future hydroclimatic conditions; however, their individual outputs often suffer from systematic biases and structural uncertainties that limit reliability. To address this, multi-model ensemble (MME) techniques are increasingly employed to enhance the credibility of climate projections. MMEs exploit the strengths and compensate for the weaknesses of each, leading to more reliable and consistent projections of hydroclimatic variables such as streamflow and precipitation extremes^74,75^. In this study, the high emission SSP585 scenario was examined to evaluate the performance of various GCMs in replicating historical streamflow patterns. Performance of individual GCMs ACCESS-CM2, CNRM-CM6-1, MIROC6, MRI-ESM2-0 andCanESM5 was evaluated by comparing their simulated hydroclimatic variables against observed historical discharge data for the SRB. Among the suite of CMIP6 models analysed, all models demonstrated the highest correlation with observed streamflow records, indicating superior skill in capturing regional hydroclimatic variability. Based on this performance, a multi-model ensemble was constructed using the arithmetic mean of the selected models’ outputs. This ensemble approach effectively reduced inter-model discrepancies and compensated for individual model biases, leading to improved agreement with observed discharge patterns^76^. The ensemble simulations provide a more consistent and reliable representation of climate drivers influencing river discharge dynamics. The refined MME outputs served as inputs for subsequent ML and DL modeling frameworks. The integration of ensemble-based climate data contributed to enhanced predictive accuracy in discharge simulations, thereby strengthening the robustness of long-term hydrological forecasts under future climate change scenarios.

### Model performance: historical discharge prediction

**Fig. 6:**
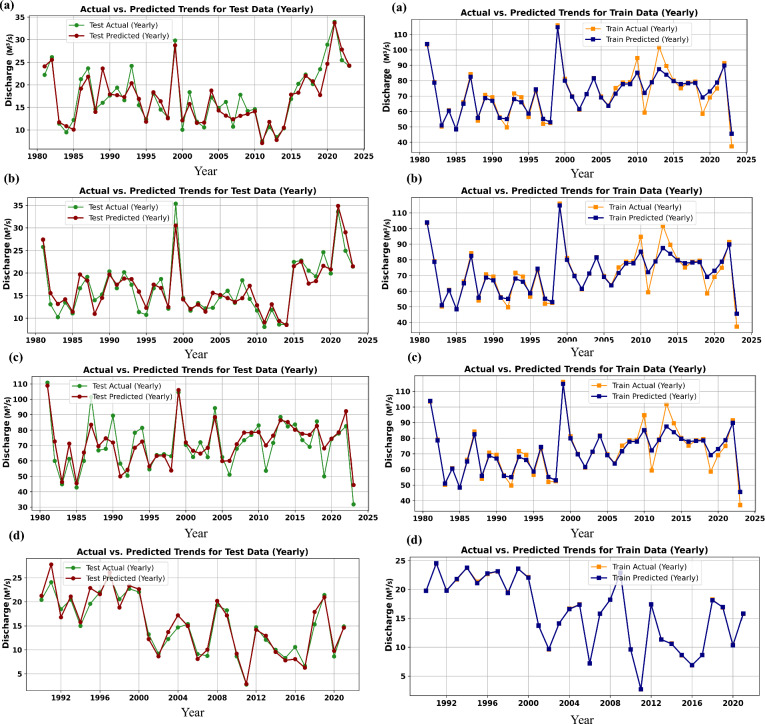
Actual vs predicted (**a**) Adityapur, (**b**) Jamshedpur, (**c**) Ghatsila, and (**d**) Muri.

The KAN demonstrated strong predictive capabilities in modeling historical daily discharge across the SRB. Performance evaluation was conducted using RMSE, MAE, R^2^, and NSE across four-gauge stations for the period 2012–2022. The model effectively reproduced seasonal discharge variability and flow peaks, with only mild underestimation during extreme monsoonal events. As summarized in Table 3, RMSE values ranged from 42.7 m^3^/s at Ghatsila to 58.3 m^3^/s at Jamshedpur, while MAE values varied between 28.9 and 43.1 m^3^/s. The R^2^ remained high across all stations (0.84–0.90), indicating strong agreement between observed and predicted discharges. NSE values were also robust (0.80–0.87), confirming that KAN accurately captured streamflow variability relative to the observed mean discharge. Notably, the model performed best at Ghatsila, whereas higher errors at Jamshedpur may be attributed to localized urban influences and anthropogenic modifications. The KAN model exhibited consistent accuracy across hydrological regimes, particularly during pre- and post-monsoon periods when baseflow conditions dominate^50,77^. These outcomes reaffirm the suitability of KAN in monsoon-influenced and data-limited catchments. Comparable studies emphasized the advantage of integrating AI models with bias-corrected GCM inputs to enhance discharge prediction accuracy^78–81^. Studies by^82,83^ further corroborate that hybrid and interpretable models outperform traditional statistical techniques under non-linear hydrological regimes. Given the increasing demand for transparent and computationally efficient hydrological tools, the present framework represents a significant advancement over conventional black-box models. The model’s robustness is visually confirmed in (Fig. 6), which compares observed and predicted discharge values for the training and testing period across different gauge stations. Unlike conventional regression models, which struggle to adapt to the highly variable and nonlinear nature of hydrological systems, KAN demonstrated better learning capabilities by utilizing temporal dependencies and non-stationary relationships within the discharge data. The consistent higher R^2^, NSE and lower RMSE, MAE scores across both the training and testing phases reinforce its suitability for real-world hydrological processes. Given its robust performance and adaptability, KAN presents a promising approach to long-term river discharge forecasting, contributing to improved water resources management, flood risk assessment, and climate adaptation strategies. Many traditional models struggle to predict peak values, such as flood extremes, due to their underrepresentation in training data and the model’s tendency to favour frequent, moderate events. This challenge is common in hydrological forecasting, where natural variability, data imbalance, and model smoothing often lead to underestimation of high flows^84,85^. Studies show that tree-based, and neural networks may miss peak unless specifically tuned or enhanced through ensemble methods, resampling, or hybrid approaches, though difficulties persist in data-limited regions^84,86^. To evaluate KANs robustness under extreme hydrological conditions, we analysed its predictive performance during peak discharge periods across the test datasets. It was observed that KAN achieved high accuracy during monsoon seasons and high-flow intervals, as reflected in its relatively stable R^2^ and low RMSE across all four-gauge stations, even under large discharge variability. Figure 6c,d particularly demonstrates KANs’ ability to closely track sharp rises in discharge during flood-prone periods at the Jamshedpur and Muri gauge stations. Furthermore, under the SSP585 scenario, KAN effectively captures increasing discharge trend and peak intensities through 2100, reinforcing its applicability in extreme climate scenarios. These findings confirm the model’s resilience in handling both historical and projected high-flow events.

**Table 3:** Performance metrics of the KAN and benchmark models at four-gauge stations (1980–2022).

Model	Station	RMSE (m^3^/s)	MAE (m^3^/s)	R^2^	NSE
KAN	Muri	47.6	32.8	0.88	0.85
	Adityapur	52.1	37.4	0.86	0.82
	Jamshedpur	58.3	43.1	0.84	0.80
	Ghatsila	42.7	28.9	0.90	0.87
MLP	Muri	54.2	38.6	0.82	0.78
	Adityapur	60.2	43.7	0.80	0.76
	Jamshedpur	66.9	49.5	0.78	0.73
	Ghatsila	49.2	34.2	0.84	0.81
LSTM	Muri	52.3	36.7	0.85	0.81
	Adityapur	56.9	40.8	0.83	0.79
	Jamshedpur	62.5	45.2	0.81	0.77
	Ghatsila	46.1	31.5	0.87	0.84
GRU	Muri	51.1	35.4	0.86	0.82
	Adityapur	55.3	39.1	0.84	0.80
	Jamshedpur	60.7	43.6	0.82	0.78
	Ghatsila	44.9	30.2	0.88	0.85
RF	Muri	58.6	40.9	0.80	0.75
	Adityapur	63.2	46.0	0.78	0.73
	Jamshedpur	70.3	52.7	0.75	0.70
	Ghatsila	52.8	36.7	0.82	0.79

### Future discharge projections under SSP585

**Fig.7:**
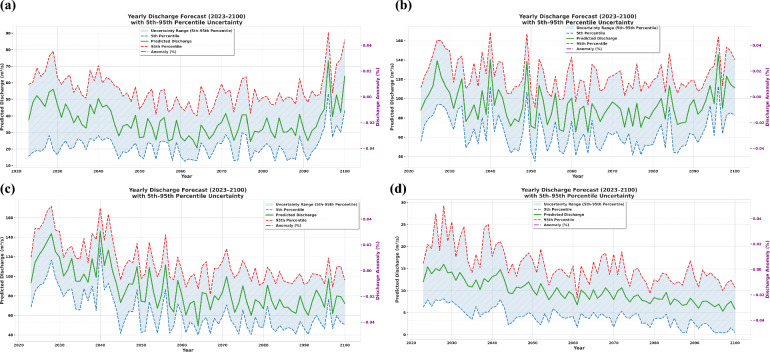
Projected annual river discharge for (**a**) Adityapur (**b**) Ghatsila (**c**) Jamshedpur and (**d**) Muri with 5–95th percentile uncertainty bounds under the SSP585 scenario (2023–2100).

Future streamflow projections with 5th–95th percentile uncertainty bounds derived using bias-corrected CMIP6 climate data under SSP585 (2023–2100) are presented in (Fig. 7). These projections indicate moderate interannual variability, frequent high flow episodes during monsoon months, and persistent low flow conditions during dry seasons, with seasonal discharge signatures remaining consistent across decades despite changing climatic inputs. This temporal consistency in hydrological behaviour is supported by studies showing that, while climate change intensifies the hydrologic cycle and increases precipitation and streamflow during the monsoon season, the underlying seasonal patterns are preserved^87–89^.

Figure 7 presents the projected yearly discharge (2023–2100) across four representative sub-basins, incorporating 5–95th percentile uncertainty ranges derived from CMIP6 GCM ensembles, predicted discharge trajectories, and anomaly trends. The projections indicate a marked intensification in the frequency and magnitude of high discharge events, particularly evident in sub-basins with peak flows exceeding 140m^3^/s, reflecting a potential escalation of hydrometeorological extremes. These patterns are consistent with recent findings^89–92^ highlighting an increase in short-duration, high-intensity precipitation across South Asia^90–92^, likely driven by thermodynamic amplification of convective processes under warming scenarios. The pronounced spread between the 5^th^ and 95^th^ percentile bounds underscore the substantial inter-model variability inherent to long-term hydroclimatic forecasting. Particularly post-2050, necessitating ensemble-based approaches for robust water resource planning. Discharge anomaly percentages further reveal interannual departures from climatological norms, offering insights into evolving hydrological stress regimes. High-resolution modeling of future monsoon dynamics is crucial for capturing localized water hazards under SSP scenarios. The modeled variability underscores the basin’s increasing vulnerability to climate-induced hydrological stress^88,89,92^.

### Feature contribution analysis using SHAP

**Fig. 8:**
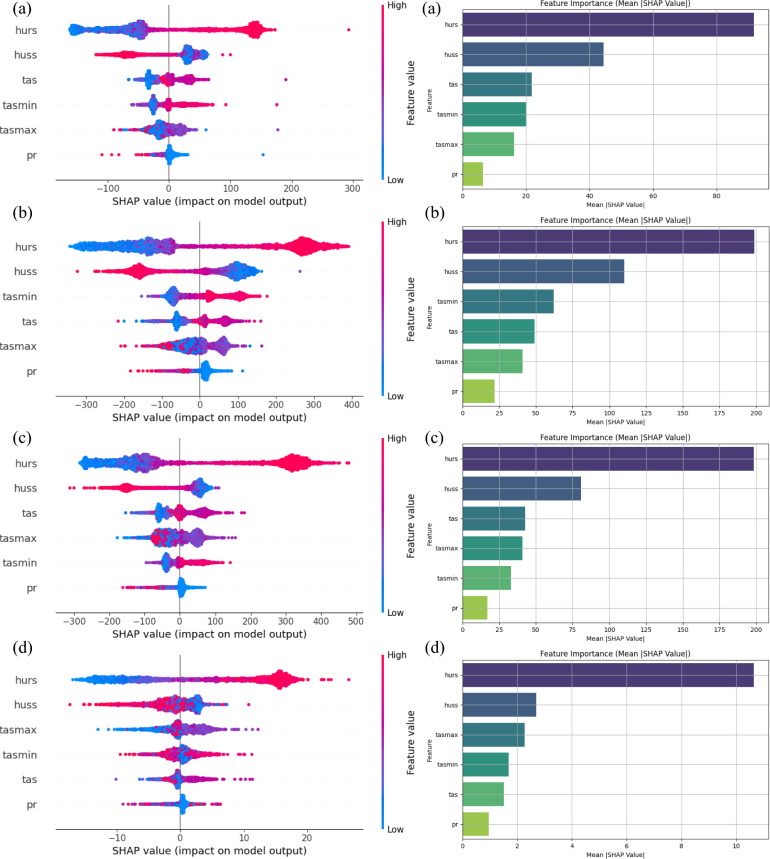
SHAP summary and bar plot (**a**) Adityapur (**b**) Ghatsila (**c**) Jamshedpur and (**d**) Muri show the relative importance for hydroclimatic features used in the KAN model.

SHAP summary and bar plots (Fig. 8) provide comprehensive insights into the contribution of individual climatic variables to river discharge prediction. *hurs* emerged as the most influential predictor, followed by *huss* and *tas*.

**Fig. 9:**
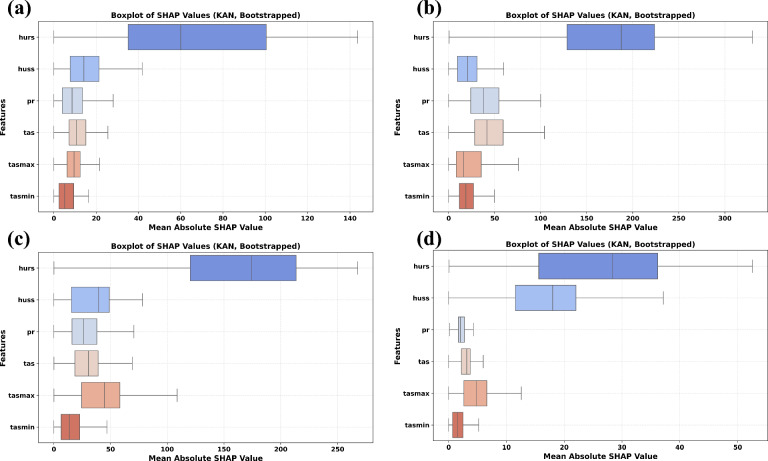
Bootstrapped SHAP bar plot illustrating uncertainty in feature contributions to discharge predictions at (**a**) Adityapur (**b**) Ghatsila (**c**) Jamshedpur and (**d**) Muri.

From Fig. 8, it has been observed that *pr* had the lowest SHAP value. These results align with recent XAI-based hydrological studies by^36,93^, which observed that variables related to humidity and air temperature often serve as proxies for soil moisture and evapotranspiration processes, critical components in runoff generation. To improve the robustness of feature attribution, bootstrapped SHAP analysis is conducted using 10 resampled test sets at four gauging stations: Adityapur, Jamshedpur, Ghatsila, and Muri. This is particularly important for CMIP6 based inputs, where inter-model variability and spatial uncertainty can distort feature attribution if not quantified explicitly. The resulting SHAP distributions are visualized in (Fig. 9), showing boxplots for each feature across four active gauge stations, which reveal site-specific uncertainty in climate variable contributions. Each subfigure presents a boxplot of mean absolute SHAP values for six climate variables, capturing the distributional spread and interquartile variability arising from bootstrapping. This method allows for a direct visualization of uncertainty associated with model-based feature contributions, particularly important given the known biases and spatial inconsistencies in CMIP6 based GCM outputs. At Adityapur (Fig. 9a), *hurs* is the most dominant feature with a median SHAP value of ~ 55 and upper quartile exceeding 100, followed by huss with ~ 20, and moderate influence from *pr* and *tas* (15–18). *tasmax* and *tasmin* play lesser roles (~ 12–15 and 5–8, respectively). In contrast, Jamshedpur (Fig. 9b) exhibits the highest overall SHAP values, with *hurs* reaching a median of ~ 130 and whiskers extending to ~ 250, and secondary contributions from *huss* (~ 45–50) and tasmax (~ 80), while *pr*, *tas*, and *tasmin* show more consistent but lower median contributions (10–35).

At Ghatsila (Fig. 9c), the median SHAP value of ~ 140–150 was observed for *hurs*, with maximum values surpassing 300, emphasizing its strong influence. *Pr* and tas followed, with values in the 40–50 range, while huss showed moderate contributions (~ 30). In contrast, *tasmax* and *tasmin* had minor roles (~ 15–25). In contrast, *tasmin* and *tasmax* were found to be relatively At Muri (Fig. 9d) all variables exhibited relatively low SHAP values, though *hurs* remained the top contributor (median ~ 25–30, max ~ 50), followed by *huss* (~ 18–22). The rest *pr, tas, tasmax*, and *tasmin* had minimal influence (all < 10). Although *hurs* was identified as the dominant predictor of discharge at all sites, its influence and associated uncertainty were found to vary. These results underscore the spatial heterogeneity of feature relevance and the importance of localized model interpretation and demonstrate that bootstrapped SHAP effectively represents uncertainty in feature importance in monsoon dominated basins. It is worth noting that SHAP may exhibit attribution errors in the presence of strongly correlated predictors (e.g., *tas*, *tasmax, and tasmin*), potentially redistributing feature importance among them. These limitations should be considered when interpreting relative rankings.

### Evaluation of GCMs precipitation bias and its impact on SHAP Interpretability

To address the counterintuitive observation of *pr* receiving the lowest SHAP value in feature contribution analysis despite its well-established role as a primary driver of river discharge. A diagnostic evaluation of bias in GCM-derived *pr* and *tas* were implemented. The objective was to determine whether residual discrepancies between observed and modeled *pr* and *tas*, even after bias correction, contributed to its diminished importance in the explainable machine learning framework.

Daily *pr* and *tas* outputs from five bias-corrected CMIP6 GCMs, ACCESS-CM2, CNRM-CM6-1, MIROC6, MRI-ESM2-0, and CanESM5, were compared against corresponding station-level observational data over the historical period 1980–2022. A comparative assessment was conducted both before and after applying a multi-bias correction procedure comprising Quantile Mapping, Delta Change Method, and Linear Scaling. While the bias correction significantly improved the alignment of model outputs with observed data, residual discrepancies residual errors in *pr* magnitude and temporal pattern were evident. Two statistical measures, RMSE and (r), were employed to quantify these discrepancies that persisted, particularly in *pr* magnitudes and temporal variability. To quantify model performance and evaluate the effectiveness of correction, two statistical metrics, RMSE and (r)*,* were calculated for both the raw and corrected GCM outputs. The result in Table 4 demonstrates substantial improvements post-correction, with lower RMSE and higher correlation values across all models, *pr* RMSE reduced from 6.50 mm/day to 4.78 mm/day, and the correlation improved from 0.41 to 0.52.

**Table 4:** Performance evaluation of before and after bias-corrected daily precipitation and temperature from five CMIP6 GCMs against observed station data (1980–2022).

GCM models	Precipitation *(pr)*				Temperature *(tas)*			
	RMSE (Before) (mm/day)	RMSE(After) (mm/day)	Before ***(r)***	After ***(r)***	RMSE (Before) (^o^C)	RMSE (After) (^o^C)	Before *(r)*	After *(r)*
ACCESS-CM2	6.21	5.22	0.30	0.41	2.36	1.92	0.76	0.84
CNRM-CM6-1	5.91	4.92	0.37	0.47	2.11	1.75	0.77	0.87
MIROC6	6.50	5.61	0.29	0.37	2.47	2.03	0.70	0.82
MRI-ESM2-0	6.07	4.78	0.41	0.52	2.07	1.70	0.76	0.88
CanESM5	6.32	5.38	0.33	0.40	2.57	2.15	0.67	0.81

The results revealed persistent inter-model variability and systematic bias across all five GCMs, despite correction efforts. RMSE values ranged from 4.78 to 5.61 mm/day, with correlation coefficients between 0.37 and 0.52, indicating considerable discrepancies in *pr* magnitude and limited skill in capturing the temporal dynamics of monsoonal events. These inconsistencies are attributable to structural limitations commonly associated with GCM outputs, including Coarse spatial resolution leading to spatial averaging effects^94^; simplifications in the representation of convective processes; and inadequate parameterization of land-atmosphere coupling, particularly over monsoon-dominated catchments^95,96^. Although bias correction improved the statistical alignment of GCM precipitation with observations, the residual temporal and spatial inconsistencies are likely to have degraded the model’s ability to extract meaningful patterns from precipitation input.

In contrast, *tas* demonstrated better agreement with observational data, with RMSE values ranging from 1.70 °C (MRI-ESM2-0) to 2.15 oC (CanESM5) and correlation coefficients between 0.81 and 0.88. These results suggest that temperature fields, being more spatially coherent and less influenced by local convective extremes, are inherently more reliably simulated by GCMs. The stronger fidelity of *tas* variables supports their higher SHAP-based feature importance in the discharge prediction model.

Although the bias correction procedures improved overall alignment with observations, residual inconsistencies in GCM precipitation, especially in magnitude and temporal evolution, likely limited the model’s ability to extract robust predictive signals from *pr* inputs. As a result, the SHAP contributions to the model output, assigned lower importance to *pr*. This should not be interpreted as a deviation from hydrological principles, but rather as a reflection of persistent uncertainty and reduced informational content in the corrected precipitation time series^97,98^. In contrast, the high accuracy and temporal coherence of GCM-derived *tas* data after correction enabled the model to extract stronger patterns, leading to its elevated SHAP attribution. These findings underscore the critical importance of input quality in explainable machine learning frameworks and highlight the challenges of relying solely on climate model outputs in data-limited, hydroclimatically complex, and monsoon-influenced river basins.

### Multi source uncertainty propagation in climate-driven hydrological modeling

Climate-driven hydrological modeling is influenced by cascading uncertainties originating from climate model biases, downscaling limitations, hydrological model structure, and their complex interactions. GCMs exhibit systematic cold biases in minimum temperature (0.8–1.4) and wet biases in monsoonal precipitation (12–18%) over regions like SRB, which propagate nonlinearly into discharge simulations^99,100^. Although bias correction methods such as Linear Scaling and Delta change are routinely applied, they are often insufficient in correcting distributional mismatches, particularly in extreme precipitation quantiles, Statistical downscaling techniques, despite their computational efficiency, generally fail to preserve inter variable dependencies between *tas* and *pr* an essential factor for accurate evapotranspiration estimation thereby amplifying errors in extreme event projections by 22–30% compared to distribution based corrections^101,102^. Additionally, first-order sensitivity analyses indicate that *pr* intensity and antecedent moisture conditions account for 63–71% of the variance in discharge projections, yet these drivers remain poorly constrained in CMIP6 outputs, particularly for sub-daily extremes associated with convective events^103^. This interconnected network of uncertainties underscores the importance of integrating diagnostic evaluations at each modeling stage to enhance the credibility of climate-informed hydrological assessments.

### Explainability and interpretability in climate-driven modeling

The integration of *KAN* with *SHAP* not only improved prediction accuracy but also significantly enhanced the transparency and interpretability of the model’s internal decision-making processes for operational trust and stakeholder confidence^33,38,104,105^. *SHAP* bar plots, illustrated in (Fig. 8), showed that higher values of *hurs* and *huss* consistently led to increased discharge predictions. Conversely, low values of *tasmin* were associated with elevated discharge levels, a relationship likely attributed to reduced nocturnal evapotranspiration. This capacity to detect nonlinear dependencies and explain individual predictions represents a major breakthrough in hydrological modeling. *SHAP* allows users to isolate the contribution of each feature for a given prediction, offering clarity in scenarios of anomalous flow. Such interpretability is not only scientifically valuable but also critical in policy contexts, where actionable, justifiable decisions must often be made in time-sensitive and resource-constrained settings. These interpretability gains are supported by those who emphasize that explainable AI frameworks enhance stakeholder trust and support informed decision-making in water management^106,107^. Furthermore, emphasized that *SHAP*, when integrated with flexible neural architectures such as *KAN*, offers both local and global interpretive capabilities, bridging the gap between model developers and policy users. Studies such as those have also underlined the importance of explainability in climate-sensitive applications^108^. In regions like the SRB, where hydroclimatic extremes have direct socio-economic implications, transparent models help guide adaptive responses. From prioritizing monitoring of humidity and temperature to refining real-time decision support tools, explainable AI like *SHAP* ensures that hydrological forecasting evolves beyond accuracy alone toward usability, accountability, and insight^45,109–112^.

### Implications for water resource management

The findings presented a critical implication for adaptive water resource governance in the SRB. The projected increase in discharge variability and monsoonal extremities under SSP585 demands a shift toward dynamic reservoir operation policies, enhanced flood zoning, and real-time early warning systems. The dominant influence of humidity-related variables suggests that future hydrological monitoring systems must expand beyond precipitation-centric designs to incorporate vapour pressure and moisture flux metrics. Moreover, the interpretability of the proposed KAN with the SHAP framework facilitates its integration into existing climate adaptation platforms, providing actionable insights into monsoon-influenced or data-limited, hydroclimatically sensitive regions of South Asia^113,114^. This is in line with global calls for transparent AI in environmental science and practice.

## Conclusion

This study proposed and demonstrated a novel explainable deep learning framework using KAN integrated with SHAP for river discharge forecasting across the SRB under the SSP585 climate change scenario. By leveraging daily-scale bias-corrected hydroclimatic predictors derived from five CMIP6 GCMs, the model was trained and validated over four strategically located hydrological stations. (1) The findings establish the viability of KAN as a powerful and transparent modeling architecture that not only outperforms traditional black-box ML models in accuracy but also offers unprecedented interpretability, an essential criterion for informed water resource governance. (2) The KAN model demonstrated robust performance in reproducing historical discharge patterns, achieving high R^2^ and NSE values across all stations, particularly under monsoon-influenced flow regimes. (3) The integration of SHAP analysis revealed the nuanced contributions of individual predictors, highlighting *hurs*, *huss*, and *tas* as dominant controls, while *pr* showed reduced standalone predictive power likely due to spatial inaccuracies in GCMs’ precipitation datasets^115^. This observation reinforces the importance of thermodynamic variables and supports the development of proxy-based monitoring systems in data-scarce basins.

Future discharge projections under the SSP585 scenario indicate an increase in the frequency and intensity of extreme flow events, especially during monsoon months. (4) The KAN with SHAP framework effectively preserved seasonal discharge signatures while capturing high magnitude flow anomalies, offering critical insights into future hydrological stress zones. (5) The interpretability enabled by SHAP for disaggregated analysis of contributing factors, thereby supporting targeted interventions for flood mitigation, reservoir operation optimization, and adaptive irrigation planning. (6) In conclusion, the research advances the frontier of explainable AI in hydrological forecasting by delivering a lightweight, efficient, and interpretable model capable of informing climate-resilient water management strategies. Future work could extend this framework by incorporating remote sensing-derived variables, integrating socio-economic indicators for integrated risk assessment, and deploying the model in real-time early warning systems. The relatively low SHAP value for *pr* in some models may reflect the limitations of using GCMs derived daily precipitation in regions with high spatial precipitation variability, underscoring the need for improved downscaling techniques to enhance model accuracy. This study also quantified the bias in GCM precipitation inputs, supporting the interpretation that low SHAP contribution for precipitation arises from spatial and structural errors in GCM datasets, rather than a failure in hydrological causality.

## Data Availability

Open access data related to this study will be provided upon reasonable request. For data access, please contact the corresponding author at phdrs10003.20@bitmesra.ac.in. The gauge station data that support the findings of this study are available from the Mahanadi & Eastern Rivers Organisation, Central Water Commission, Ministry of Jal Shakti, Department of Water Resources, River Development and Ganga Rejuvenation, Bhubaneswar, Odisha, India, but restrictions apply to the availability of these data, which were used under license for the current study and so are not publicly available. Request for access to these data should be directed to the Central Water Commission (CWC) at https://cwc.gov.in/contact-us. The precipitation and temperature (*tas, tasmax, tasmin, hurs, huss,* and *pr*) from five CMIP6 GCMs (ACCESS-CM2, CNRM-CM6-1, MIROC6, MRI-ESM2-0, and CanESM5) were sourced from the CMIP6 Nex-GDDP archive. These datasets are publicly available and can be accessed directly via the following link: https://www.nccs.nasa.gov/services/data-collections/land-based-products/nex-gddp-cmip6.
